# Animal-Assisted Interventions and Animal Welfare—An Exploratory Survey in Germany

**DOI:** 10.3390/ani13081324

**Published:** 2023-04-12

**Authors:** Katharina Ameli, Theresa F. Braun, Stephanie Krämer

**Affiliations:** 1 Interdisciplinary Centre for 3Rs in Animal Research (ICAR3R), Justus-Liebig-University, 35392 Giessen, Germany; stephanie.kraemer@vetmed.uni-giessen.de; 2 Department of Special Education and Inclusive Education, Justus-Liebig-University, 35394 Giessen, Germany; theresa.f.braun@erziehung.uni-giessen.de

**Keywords:** human–animal studies, animal-assisted therapy, animal-assisted pedagogy, animal-assisted activities

## Abstract

**Simple Summary:**

Animals play a crucial role in social occupational fields. The positive effects of animals are described in theory and practice. However, the significance of animal welfare in animal-assisted intervention settings has not yet been extensively researched. In the present project, individual views on animal welfare are analyzed based on interviews with professionals working with animals. As a result, it can be stated that animal welfare plays an important role for those working in animal-assisted interventions. The structure and design of assignments, animal-related aspects, and conditions as well as education and knowledge are mentioned as generally relevant conditions for compliance with animal welfare from the perspective of animal-assisted intervention practitioners. In addition, concrete courses of action to ensure animal welfare are described. These include stopping or changing the setting at different levels. It is not possible to assess the actual implementation of animal-assisted intervention settings in the context of animal welfare. Further studies are needed to analyze this.

**Abstract:**

Background: Animals play a crucial role in social occupational fields. The positive effects of animals are described in theory and practice. However, the significance of animal welfare in animal-assisted intervention settings has not yet been extensively researched, so that the aim of this explorative study was to investigate the perception and significance as well as the understanding of animal welfare and its implementation on the part of professionals working with animals. Methods: In the present project, 270 animal-assisted professionals from Germany were interviewed about their individual perceptions of animal welfare and their implementation of animal welfare with the help of questionnaires with closed questions (5-point agreement scale) and open questions. The quantitative data were analyzed using the statistical software SPSS and MS Excel. The qualitative data were analyzed using thematic coding. Results: The quantitative and qualitative results show that animal welfare poses high importance for people working in animal-assisted interventions. The structure and design of assignments, animal-related aspects and conditions, and education and knowledge are mentioned as generally relevant conditions for ensuring animal welfare from the perspective of animal-assisted intervention practitioners. In addition, different concrete courses of action to ensure animal welfare are described, which are characterized as stopping or changing the setting at different levels. Conclusions: Animal welfare plays a central role for professionals working with animals. However, further studies are necessary in order to record other animal welfare-relevant aspects in the animal-assisted interventions, depending on the respective animal species, and to examine the implementation of animal welfare-related measures.

## 1. Introduction: Animal Welfare in Animal-Assisted Interventions

The human–animal relationship manifests itself in different facets and cultural contexts [1,2,3]. In this context, animals are assigned different roles by humans, which vary, for example, between the perception of animals as products and partners in everyday life [4]. One form of this human–animal relationship is the targeted use of different animal species in social fields of action. The variety and range of animal species used are large. For example, dogs, horses, and donkeys, but also chickens, New World camelids, guinea pigs, agate snails, and bees are used in educational or therapeutic contexts.

In Germany, the field of animal-assisted activities is largely subsumed under the term animal-assisted interventions. Going back and following the Delta Society (today Pet partners), a subdivision is made in Germany into animal-assisted therapy, animal-assisted pedagogy, animal-assisted activities, and animal-assisted support measures [5,6]. In addition, other terms are used that are more specific to the animal(s) used, such as dog-assisted pedagogy in schools [7] or equine-assisted coaching in human resource development [4,8]. So far, statistical data on the number of concrete animal-assisted services in Germany do not exist, nor does information on the distribution of the respective types of animals within the variety of services.

It should also be noted that there is no uniform use of the terms in relation to the areas of activity. However, the terms have in common that they are understood as goal-oriented professionalized animal-assisted interactions that aim at improvements in therapeutic or educational areas of activity and include the use of a (specially trained) animal.

Regardless of the specific setting of the animal-assisted interventions, there are usually three actors involved in the interactions: the professional working with the animal, the animal used, and the target person. Furthermore, there are additional persons who support the person carrying out the intervention. This happens, for example, by leading an animal or supporting larger groups or groups with special needs. The actors or groups of actors involved move in the specific situation in an interaction triangle (triad) (see also [6]). This triad shows that the human–animal interactions in animal-assisted interventions consist of several dimensions that are reciprocal and dynamic in different directions, increasing the complexity of the same [9,10]. The triadic connection among the people, the animals and the clients, is based on a professionalized interaction. The practitioners, the animals, and the clients can communicate and participate equally. Thus the triadic relationship is adaptable.

In the triadic interaction, the professional practitioners play the most significant role of all actors involved. They need to reflect on the professional actions, which are ensuring animal welfare and the well-being of the clients in their specific role. Nevertheless, the animal agency must be considered to create a professional setting. The majority of current research in animal-assisted interventions shows a focus on the positive effects of the animal species used on humans on a physical, psychological, or social level (see also [6,11,12,13,14,15,16]). On the other hand, the reverse effect, i.e., the effects of the humans involved on the animals, has not yet been scientifically investigated. However, it is generally known that animals, just like humans, can feel stress and strain [17,18,19,20,21]; thus, issues of ensuring animal welfare have also become relevant to the context of animal-assisted interventions.

Based on the German Animal Welfare Act, animal welfare can be characterized as the responsibility of humans for animals as fellow creatures whose lives and well-being must be protected. No one may inflict pain, suffering, or harm on an animal without reasonable cause [22]. Pain is referred to as unpleasant sensory and emotional experiences related to actual or potential tissue damage [23]. Suffering, on the other hand, occurs when an animal is not able to satisfy its needs, meet its needs, and/or avoid harm with its species-typical behavior, because it must be assumed that it experiences inadequate coping abilities in such situations. Suffering becomes comprehensible for the observer when clear and/or longer-lasting deviations from normal behavior can be observed [24]. Harm here means that the animal’s physical and mental state is temporarily or permanently altered for the worse. The death of the animal is also considered to be harm, and harm is often preceded by suffering [23].

Following the One Health Approach [25] and the One Welfare Approach [26], it should be emphasized that the well-being of animals poses a significant role to participate positively in human–animal interactions. To ensure this, a high level of professional behavior and consideration of the agency of the animal is required.

In conclusion, this means that pain, suffering, and harm must be avoided in animal-assisted interventions. The resulting moral obligation of animal-assisted professionals toward animals involves safeguarding both the well-being and life of the animals in animal-assisted interventions. Well-being can be characterized as a construct that varies according to discipline and context. In the sense of Pollmann and Tschanz [24], for the animal-assisted field, the well-being refers to a state of physical and psychological harmony of the animal within itself and with the environment, which is characterized in particular by freedom from pain and suffering [23]. For this reason, the display of normal behavior is also identified as an essential marker of well-being. People who offer animal-assisted interventions must therefore firstly have a high level of ethological knowledge of the respective animal species and its needs. Secondly, a focus on the individual is important to ensure the well-being of the animal, but also to prevent pain and suffering [17,27]. In the specific case of animal-assisted interventions, this means noticing possible signals of suffering and/or pain from an animal during an interaction and taking appropriate action to minimize, resolve, or end the animal’s suffering and/or pain. This includes removing the causes or terminating the interaction to ensure the animal’s well-being. In order to strengthen animal welfare in animal-assisted interventions and to ensure the well-being of animals, Germany has anchored a relevant test criterion in Section 11 of the German Animal Welfare Act, which is uniform nationwide.

The German Animal Welfare Act is nationally valid and applicable to all animals. Nevertheless, there is room for interpretation of the law, which allows the 16 federal states to apply the law in different ways.

This means that each veterinary office independently regulates the conditions under which it gives permission.

For animal-assisted professionals, this means that they must apply for a certificate of competence from the relevant veterinary authority if they work commercially and:

- breed or keep vertebrate animals, except farm animals and wild game;
- maintain a riding or driving operation;
- exhibit animals, or make them available for such purposes [22].

A commercial activity within the meaning of the German Animal Welfare Act is deemed to exist if the activities mentioned are carried out independently and in a planned manner and with the intention of making a profit. Pursuant to Article 11 No. 5 of the German Animal Welfare Act, the practice of commercial animal-assisted activities may only be started after the competent authority has granted the license. Within the framework of a technical discussion, relevant knowledge of the animal species used is examined. In addition, sufficient skills and abilities in the practical handling of the animal species in question must be demonstrated. Finally, the authority checks the facility where the animals are kept or where the animal-assisted activity is carried out. Depending on the conditions, a permit can also be issued for a limited period of time or subject to conditions. Finally, the permit can be withdrawn in case of violations of the German Animal Welfare Act.

Concluding from the legal, but also moral provisions, it has been shown that a professional working with animals has the most important role in the interaction triangle. This is based on the assumption that they are equally responsible for all actors and that they have a decisive influence on events through their existing theoretical and practical knowledge of the animal (e.g., species-specific needs and expressive behavior), target group (e.g., developmental steps and disorders), and profession-related methods and concepts. An analysis of the importance of animal welfare and its concrete implementation in practice is currently still a desideratum. Important questions on the animal perspective and, consequently, the guarantee of animal welfare have not been conclusively analyzed for animal-assisted interventions [28]. Missing data represent a research problem at the interface between animal-assisted interventions and animal welfare.

This is where the present study comes in. With the help of questionnaires for professionals working with animals, we will find out what role animal welfare plays in animal-assisted interventions from the point of view of professionals and which characteristics for its implementation they consider to be particularly important. It addresses two overarching research questions: (1) What importance do practitioners assign to animal welfare in animal-assisted interventions? (2) What criteria are named to ensure animal welfare in animal-assisted interventions?

## 2. Materials and Methods

### 2.1. Objective

The aim of this explorative study was to examine the perception, importance, and understanding of animal welfare and its implementation on the part of professionals working with animals. The formulation of the hypotheses as a scientific theory-based statement was based on the following hypothesis in relation to the theory and the qualitative results: “Animal welfare plays a major role in animal-assisted interventions, although its implementation in practice varies”.

### 2.2. Survey Method

The survey implemented here with the help of a questionnaire was preceded by a detailed problem definition through the elaborated theoretical analysis of existing studies and theoretical receptions in the topic area of animal-assisted interventions and animal welfare. Only after all the details of interest had been established was the survey instrument formulated in order to ensure operationalization [29].

The questions in the questionnaire (Supplementary Material S1) were based on a 5-point approval Likert scale. A high value means high agreement, and a low value means low agreement [30].

Due to the complexity of the topic “animal welfare and animal-assisted interventions” and the need to reflect on this, the questions were chosen in such a way that far-reaching characteristics were included in the analysis. All questions were standardized and formulated as largely closed questions. This minimized the influence of the researcher on the respondents [29]. A total of 71 variables were collected as part of the survey. In addition to the closed questions, qualitative questions on the concrete strategies in the implementation of animal welfare at animal-assisted interventions were integrated.

Before the final survey, the plausibility of the questions was checked in advance by a pretest with animal-assisted practitioners in training [29]. Following on from this, no changes were made to the survey instrument. Personal data were collected and processed in the context of this study. In addition to the ethical guidelines for conducting research, projects of the German Sociological Association (DGS) and the General Data Protection Regulation were also considered. This ensures that no data are passed on to third parties and that all personal data that could be used to draw conclusions are anonymized throughout the project [29].

### 2.3. Sampling

The present survey is based on an opportunity sample (also called ad hoc sample). This nonprobabilistic type of sampling in the form of an ad hoc sample is particularly suitable for exploratory projects, as it involves interviewing people who are available and easily accessible.

The dissemination of the questionnaire took place online via distribution lists and networks of animal-assisted intervention (professional) organizations as well as via social platforms (topic-specific groups). This allowed all persons interested in the topic to participate in the survey [31]. A total of 270 people (*n* = 270) took part in the survey.

Due to the diverse distribution of animal-assisted professionals in different fields of activity, a basic population in the form of structural data was not accessible in advance. This means that the representativeness was not quantifiable [29]. Consequently, the project makes no claim to representativeness and generalizability. The results serve as indicators for a descriptive topic-specific opinion regarding the perception and implementation of animal welfare in animal-assisted interventions [29].

### 2.4. Data Analysis

Only the complete questionnaires were considered in the evaluation of the data (*n* = 270). After completion of the data collection, the raw materials were sorted, annotated, formatted, anonymized, and cleaned, and these revised data sets were used for further analysis [31]. Attention was paid to the following criteria: completeness, uniformity, exclusion of duplicate values, appropriate treatment of values, recognition of missing values, and plausibility of the response patterns [32]. The preparation and statistical evaluation of the data (frequency tables, diagrams, etc.) were performed with the help of the statistical software SPSS and MS Excel. In the interpretation of the results, attention was first paid to the mean values that resulted from the agreement scale for the individual questions. This allowed the first descriptive results to be read.

In addition, the standard deviations were used in the second step, as this offers a key figure to describe the dispersion of the answers to individual questions. Here, the analysis focused particularly on the inhomogeneity of the answers, because, according to the thesis, there is no clear, uniform opinion, which in turn provides feedback on relevant aspects at the interface of animal-assisted interventions and animal welfare. In this context, standard deviations of over 1.0 were given special consideration. The qualitative responses (open questions) from the questionnaires were assessed using thematic coding according to Flick [33]. Thematic coding is a multistage procedure where the free responses were analyzed using an open coding system. The aim was to break up the data, conceptualize it, and put it together in a new way. This made it possible to combine the codes into similar categories [33]. The categories were sorted into superordinate categories according to proximity of content. This allowed a minimization of broader subcategories. This differentiation and clustering of the data were generated from the data—always in the context of the research question and research objective [34]—and followed a descriptive system.

## 3. Results

Overall, the results provide an approximation of the understanding of animal welfare in the animal-assisted interventions, as well as relevant aspects that follow on from this and which indicators for the implementation of the same the participants will consider to be particularly important.

### 3.1. General Description of the Animal-Assisted Professionals

In the results, the individual assignment to a specific field of activity shows that the largest proportion of respondents work in animal-assisted pedagogy (40.3%), followed by animal-assisted therapy (29. 5%), animal-assisted activity (16.5%), and animal-assisted support measures (13.7%). The most common field of activity is school, followed by youth welfare, day care centers, psychotherapy, and care for the elderly. In this context, dogs, horses, chickens, cats, and goats are used most frequently. The total number of animal species mentioned shows that each respondent uses an average of four animals. Of the respondents, a total of 74.5% work with special materials or work harnesses when they are working with their animals. On average, the animals are used for 4.5 hours per week.

The question as to whether a basic use of the animal is possible resulted in a high level of agreement on the part of the respondents (mean 4.10); however, the standard deviation (SD = 1.030) indicates a wide range in the response.

The basic occupations of the practitioners named are located particularly in the area of pedagogical occupational fields. The five most frequent occupational groups are educators, teachers, social (educational) workers and social workers, and psychologists. The respondents’ reasons for working with animals are largely described as the effect of the animals on the respective target group. In this context, the benefit for the people participating in the interaction is explicitly mentioned. Some interviewees particularly emphasized the relevance of animal-assisted interventions for specific target groups. Thus, it becomes clear that the function of animals as door openers and motivators is particularly emphasized, as exemplified by the following quote from a respondent: “The animal can open doors that humans often have closed” and “because animals are a high motivator”, as they are “always spontaneous and honest”. Furthermore, it becomes clear that one’s own experiences contribute to the fact that supporting animals in one’s own work is an essential aspect. In this context, the role of animals as “co-therapists” is also frequently referred to. Most respondents stated that they implement animal-assisted interventions as part of their main professional activity (51.58%). A total of 34.39% carry out the work on a part-time basis, and 14.03% are self-employed, full-time animal-assisted practitioners. For this, 94.8% of the respondents had completed a corresponding training, whereby the contents and scope of this training are not differentiated in more detail here. A total of 80.6% of the people also stated that the animal they used also had the appropriate training. It should be emphasized here that only the animal species dog and horse are named in the case of training of the animal.

Of the respondents, 63.35% are in possession of permit as outlined in Section 11 of the German Animal Welfare Act, while 36.65% do not have such a permit. The agreement with the question about the quality feature of a permit as outlined in Section 11 is overall rather in the high range (mean 3.71); however, the standard deviation (SD = 1.209) shows that the answers show a high dispersion.

### 3.2. Relevant Factors and Courses of Action for Animal Welfare from a Practical Perspective

The evaluation shows a very high agreement (92.2%, mean 4.87) of the respondents that animal welfare plays a crucial role in animal-assisted settings. In this context, it can be seen that most of the interviewees confirmed a high level of agreement (92.1%, mean 4.9) that they know the theory(s) of stress recognition and can apply them to their own animal (90.3%, mean 4.9). It is striking that only 36.5% of the respondents stated that they regularly film the animal in action and then evaluate it.

In this context, there is also a high level of agreement regarding the concrete use of procedures for stress reduction (mean 4.41) and the recognition of physical and psychological needs of the animals used (mean 4.91 and 4.85). It should not go unmentioned at this point that it remains unclear whether the procedures for stress reduction and the recognition of physiological or psychological needs are implemented in practice.

**Figure 1:**
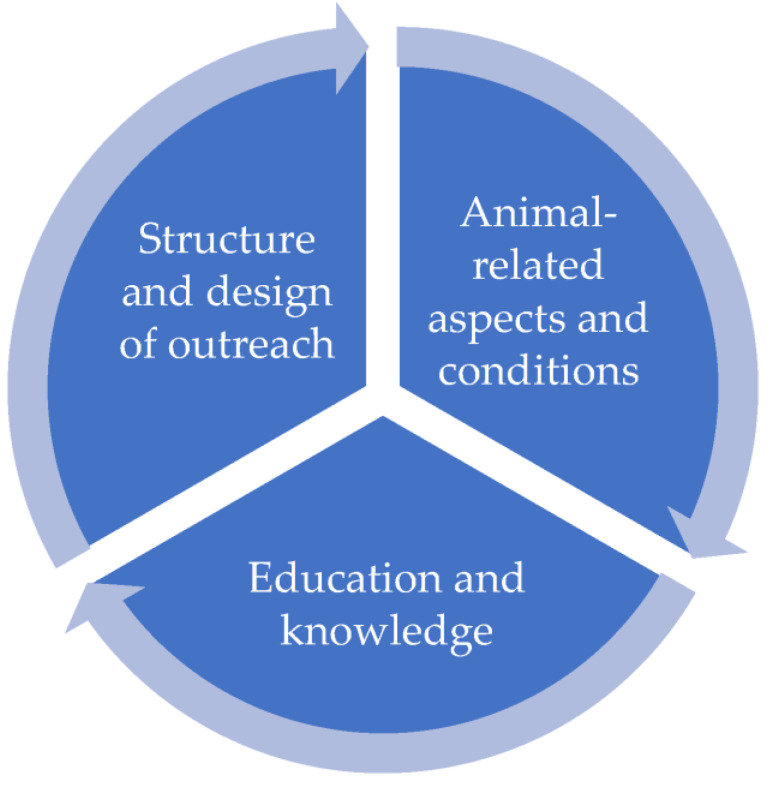
General conditions for compliance with animal welfare.

In the evaluation of the qualitative questions on the relevant conditions for compliance with animal welfare in the animal-assisted intervention settings from the perspective of the practitioners, it was possible to form various thematic clusters (see Figure 1).

The basis of the *structure and design of interventions* can be categorized as bonding. This means that there is a good relationship between animals and humans and that the animal is understood by the animal-assisted intervention practitioner as a partner in the mission. This includes that the education and training of the animal are practiced without the use of coercion and with positive reinforcement. Following on from this, the importance of the concrete planning of the respective setting is also mentioned, which should take place using suitable equipment for the animal.

The concrete design of the settings is based on voluntariness and should include breaks (mentioned across animal species) and limit the number of assignments. Finally, organizational processes are also considered relevant. These include, for example, the observance of hygiene rules for all participants or “behavioral training” with the clients.

The *animal-related aspects and conditions* make it clear that species-appropriate husbandry and care with regular veterinary checks must be ensured. The orientation of the professionals to the well-being and needs of the individual animal species, in addition to the use of healthy animals, is understood as an important marker. In this context, the abstinence from violence plays just as important a role for the interviewees as the avoidance of stress or excessive demands. Following on from this, the importance of *education and knowledge* becomes apparent, as this is seen as the basis for the implementation of animal welfare. The existence of professional competence and knowledge is considered just as important as the concrete training of humans and animals as well as the selection of suitable animal species.

**Figure 2:**
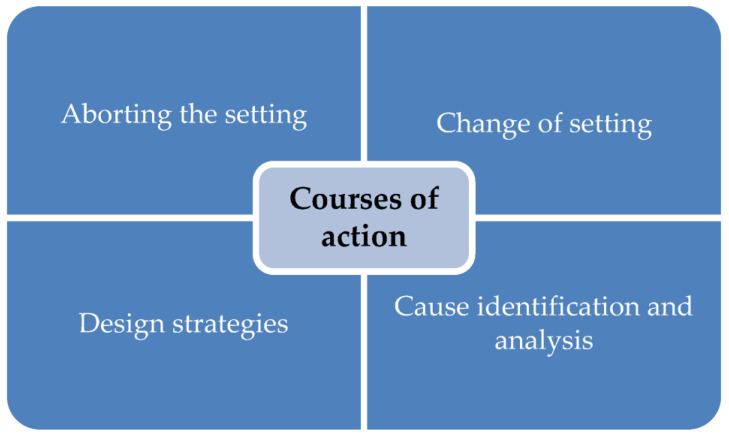
Courses of action to ensure animal welfare.

The clustering and analysis of the general conditions described above were followed in a further step by the question of concrete courses ways of action by animal-assisted intervention practitioners to ensure animal welfare across species, which is illustrated in Figure 2.

The qualitative evaluation of the actions of the animal-assisted workers shows that the most frequent measure taken by the interviewees is a change *of the setting* in order to ensure animal welfare. This stopping is characterized by taking the animal out of the situation or offering the animal a possibility to withdraw, as the following quotation shows: “I offer the animal the possibility to withdraw and explain to the client that the animal needs a break.” A joint positive conclusion before termination is also assessed as relevant. This is often followed by further work without the animals using substitute methods of the animal-assisted intervention practitioners. Sometimes the clients are also removed from the situation; however, the removal of the animal, as mentioned above, is described more frequently.

It can also be recognized within the concrete methods of action that a *change of the setting* is made on the part of the animal-assisted professionals. This includes, for example, the change of the spatial conditions or the methodical change from active to passive phases, as the following quotation from the example of the dog illustrates: “I take the dog out for a short time and offer it its place of retreat and “entertain” the clients differently.” In addition, the tasks to be implemented or the tools used to ensure animal welfare are changed in the situation. Alternatives and compensation for the animal are also considered here. The analysis also shows that the client’s behavior is perceived as a factor relevant to animal welfare, so that a change in this behavior can also be seen as a change in the setting. The change in the client’s behavior is partly accompanied by a joint reflection on the “critical” behavior regarding the triggering factors as well as finding a solution. Reflection on the situation belongs to the thematic category of *finding causes and analyzing factors* that threaten animal welfare, which was also identified. The animal-assisted intervention practitioners state that they look for the causes and the trigger of stress in the animals and try to remove them, as also indicated by the following quote: “I have to identify what exactly triggers the stress [...] and then I have to decide what I can change and how.”

In addition, certain *strategies of setting design* are used to ensure animal welfare, such as allowing all participants in the interaction to meet freely or providing safety related to the animal.

**Figure 3:**
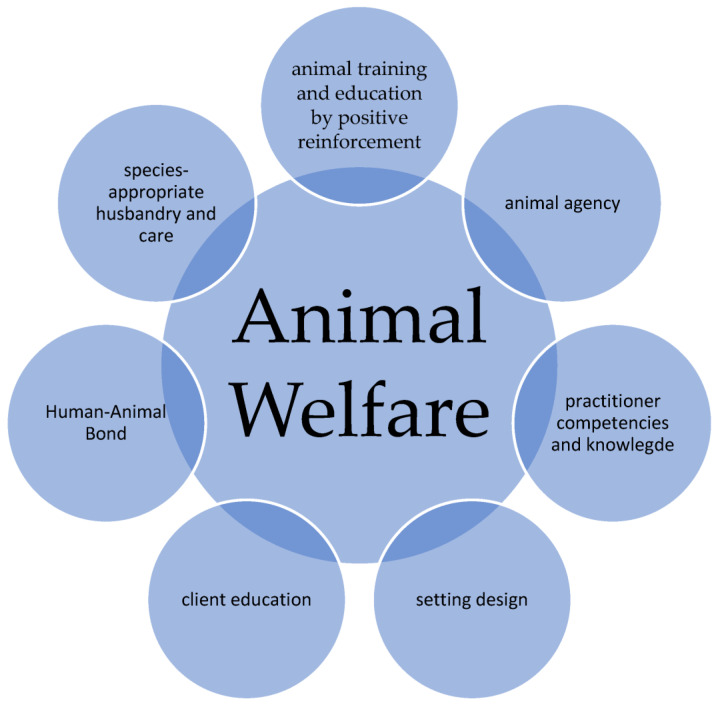
Parameters to ensure animal welfare from practitioner’s perspective.

Overall, the indicators for the implementation of animal welfare from a practitioner’s point of view can be summarized in Figure 3.

## 4. Discussion

The demographic data show that animal-assisted work is carried out in various fields of activity, especially in the educational sector. Unsurprisingly, animal welfare has an enormous significance across all fields, as the results of the survey clearly show. At the same time, it remains open whether the importance of animal welfare and the respondents’ own knowledge about the theory/theories of stress recognition and being able to apply this to their own animal(s) would correspond with an expert external assessment. The possibility of filming the animal in action and evaluating its behavior afterward, which was used by only one third of the respondents, indicates possible contradictions. Accordingly, the assessment of the well-being and possible stress behavior in the settings is based exclusively on short observation sequences in the setting itself.

It should be emphasized that it proves helpful to underpin these sequences by filming, because the complexity of animal-assisted intervention settings and interactions often exceeds human observational capacity.

Although the conclusion is speculative, it should not be disregarded that any changes in behavior take place in such a short moment, and the overall behavior cannot always be detected by the human eye at any time. Evidence to support this thesis is provided by reference to ethological studies, which are usually supported by actual video recordings in the analysis [35,36].

Another indication for the possible discrepancy between self-perception and perception by others could be the assessment of the importance of the permit mentioned in Section 11 of the German Animal Welfare Act. Obtaining this official permit is not an important feature of animal welfare compliance for all persons working in animal-assisted interventions. In addition, the permit mentioned in Section 11 of the German Animal Welfare Act—although obtained through expert discussion and peer review—is not regarded as a quality feature for animal welfare, even though aspects, such as education and knowledge as well as animal-related aspects and conditions, are to be explicitly checked by the authority, which in turn represent the three central conditional areas for ensuring animal welfare from the perspective of the interviewed practitioners. The analysis of the data also shows that the preparation and planning of the setting, which are considered highly relevant for the pedagogical field in theory and practice, do not seem to play an overriding role in the animal-assisted interventions. With a view to the triad mentioned at the beginning, a further discrepancy emerges in that the concrete planning of a setting is essential for clarifying the conditions. For example, rooms, materials, or external and internal conditions (e.g., consultation with colleagues or one’s own perception of stress) must be clarified. The documentation of the units as well as the classification of impressions, feelings, and thoughts as essential areas of implementation and follow-up do not play a role either, although this information is highly relevant for further development and adaptation possibilities.

In the context of planning and implementation, the concrete courses of action named by the interviewees to ensure animal welfare are also interesting. Thus, explicit reference is made here to the analysis; however, in the aspects named, this refers more to the concrete situation in which stress signals were perceived and less to potential parameters triggered by the setting. When observing stress signals, respondents most often react by stopping or interrupting the situation. A change in the setting is also made, such as a change in the room or a change in the methods used. At present, it remains unclear in this context which concrete factors ultimately led to stopping or which factors merely led to an adjustment of the setting. This aspect is currently being analyzed in an animal-specific follow-up study.

The chosen research method proved to be appropriate for the purpose of exploring the perception of animal welfare from the perspective of animal-assisted intervention practitioners in order to obtain a differentiated picture of opinions with concrete general courses of action to ensure animal welfare. The actual implementation of animal-assisted interventions in the context of animal welfare remains unclear, as the research design was geared toward analyzing the perspectives of professionals working in the field of animal welfare. In order to identify any discrepancies between their own perceptions of stress recognition of the animals used and the perceptions of researchers with specialized knowledge of animal behavior and welfare, a structured study comparing their own and others’ perceptions would be needed.

Regarding our hypothesis, we were nevertheless able to show overall that the importance of animal welfare plays a significant role for animal-assisted intervention professionals.

The mentioned parameters for ensuring animal welfare reveal close points of reference to professional action. From a sociological perspective, it can be argued that professional action takes place within a triad. This requires reflexive professional behavior. To ensure animal welfare in animal-assisted interventions, it must be understood as acting with a certain quality, which is ensured within the framework of concrete planning through the planning of settings. This means that precise break-off criteria are included, which are determined as soon as the setting is terminated for the animal.

## 5. Conclusions

The aim of the study was to gain a deeper understanding of the implementation of animal welfare in animal-assisted interventions. The respondents showed that they assign a high priority to animal welfare. Thus, the respondents rate themselves as being able to reliably identify animal welfare-relevant indicators in their own work and to be able to take appropriate measures to ensure animal welfare.

Due to the very low number of analyses on the implementation of animal welfare in animal-assisted interventions, we suggest that further analyses on the perception of animal welfare-relevant aspects be carried out on an animal species-specific basis.

## Data Availability

The data presented in this study are available on request from the corresponding author. The data are not publicly available due to data protection according to General Data Protection Regulation of the European Union.

## Supplementary Materials

The following supporting information can be downloaded at: https://www.mdpi.com/article/10.3390/ani13081324/s1, Supplementary Material S1, Translated Questionnaire.

## References

[1] Cyrulnik B., Matignon K.L., Fougea F. (2003). Tiere und Menschen: Die Geschichte einer Besonderen Beziehung.

[2] Borgards R. (2016). Tiere: Kulturwissenschaftliches Handbuch.

[3] Brucker R., Thieme F., Bujok M., Mütherich B., Seeliger M. (2015). Das Mensch-Tier-Verhältnis: Eine Sozialwissenschaftliche Einführung.

[4] Ameli K. (2016). Die Professionalisierung Tiergestützter Dienstleistungen.

[5] Hegedusch E., Hegedusch L. (2007). Tiergestützte Therapie Bei Demenz: Die Gesundheitsförderliche Wirkung von Tieren auf Demenziell Erkrankte Menschen.

[6] Vernooij M.A., Schneider S. (2018). Handbuch der Tiergestützten Intervention: Grundlagen, Konzepte, Praxisfelder.

[7] Beetz A. (2019). Hunde im Schulalltag: Grundlagen und Praxis.

[8] Dielmann A., Lohkamp L. (2016). Professionelles Coaching Mit Pferden. Hintergründe—Weiterbildung—Methoden. Mensch und Pferd.

[9] Ladner D., Brandenberger G. (2018). Tiergestützte Psychotherapie Mit Kindern und Jugendlichen: Hund und Pferd Therapeutisch Einbeziehen.

[10] Wohlfarth R., Mutschler B. (2017). Praxis der Hundegestützten Therapie: Grundlagen und Anwendung.

[11] Allen K., Shykoff B.E., Izzo J.L. (2001). Pet Ownership, but Not Ace Inhibitor Therapy, Blunts Home Blood Pressure Responses to Mental Stress. Hypertension.

[12] Beetz A., Uvnäs-Moberg K., Julius H., Kotrschal K. (2012). Psychosocial and Psychophysiological Effects of Human-Animal Interactions: The Possible Role of Oxytocin. Front. Psychol..

[13] Beetz A., Wohlfarth R., Kotrschal K., Beetz A., Riedel M., Wohlfarth R. (2021). Die Mensch-Tier-Beziehung und Wirkmechanismen. Tiergestützte Interventionen: Handbuch für die Aus- und Weiterbildung.

[14] Headey B., Na F., Zheng R. (2008). Pet Dogs Benefit Owners’ Health: A “Natural Experiment” in China. Soc. Indic. Res..

[15] Julius H., Beetz A., Kotrschal K., Turner D.C., Uvnäs-Moberg K. (2014). Bindung zu Tieren: Psychologische und Neurobiologische Grundlagen Tiergestützter Interventionen.

[16] Wells D. (2009). The Effects of Animals On Human Health and Well-Being. J. Soc. Issues.

[17] Bohnet W., Otterstedt C., Rosenberger M. (2012). Ethologie: Die Bedürfnisse Der Tiere in der Mensch-Tier-Beziehung. Gefährten—Konkurrenten—Verwandte: Die Mensch-Tier-Beziehung im Wissenschaftlichen Diskurs.

[18] Hornung J., Dulleck A.S., Ameli K., Dulleck A., Brüsemeister T. (2016). Der Einfluss Tiergestützter Dienstleistungen auf das Wohlbefinden des Tieres. Grundlagen Tiergestützter Dienstleistungen: Tiergestützte Therapie, Pädagogik und Fördermaßnahmen als Interdisziplinäres Arbeitsfeld.

[19] Glenk L.M. (2017). Current Perspectives on Therapy Dog Welfare in Animal-Assisted Interventions. Animals.

[20] Scopa C., Contalbrigo L., Greco A., Lanatà A., Scilingo E.P., Baragli P. (2019). Emotional Transfer in Human-Horse Interaction: New Perspectives on Equine Assisted Interventions. Animals.

[21] Meers L.L., Contalbrigo L., Samuels W.E., Duarte-Gan C., Berckmans D., Laufer S.J., Stevens V.A., Walsh E.A., Normando S. (2022). Canine-Assisted Interventions and the Relevance of Welfare Assessments for Human Health, and Transmission of Zoonosis: A Literature Review. Front. Vet. Sci..

[22] Bundesministerium der Justiz (BMJ) (2006). Tierschutzgesetz (TierSchG).

[23] Hirt A., Maisack C., Moritz J., Felde B. (2023). Tierschutzgesetz.

[24] Pollmann U., Tschanz B.L. (2006). Ein Begriff aus dem Tierschutzrecht. Amtstierärztlicher Dienst Lebensm..

[25] Mackenzie J.S., Jeggo M. (2019). The One Health Approach-Why Is It So Important?. Trop. Med. Infect. Dis..

[26] Bourque T. (2017). One Welfare. Can. Vet. J..

[27] International Association of Human-Animal Interaction Organizations (Iahaio) (2018). IAHIO Weißbuch: Definitionen der IAHIO für Tiergestützte Interventionen und Richtlinien für das Wohlbefinden der Beteiligten Tiere.

[28] Krämer S., Ameli K. (2022). Gasteditorial: Tierschutz im Fokus—Ein kritischer Blick auf tiergestützte Interaktionen. Tierethik.

[29] Braunecker C. (2021). How to do Empirische Sozialforschung: Eine Gebrauchsanleitung.

[30] Schnell R., Hill P.B., Esser E. (2011). Methoden der Empirischen Sozialforschung.

[31] Döring N., Bortz J. (2016). Forschungsmethoden und Evaluation in den Sozial- und Humanwissenschaften.

[32] Schendera C.F.G. (2007). Datenqualität Mit SPSS.

[33] Flick U. (2017). Qualitative Sozialforschung: Eine Einführung.

[34] Kuckartz U. (2014). Mixed Methods: Methodologie, Forschungsdesigns und Analyseverfahren.

[35] Kappeler P.M. (2020). Verhaltensbiologie.

[36] Ng Z.Y., Pierce B.J., Otto C.M., Buechner-Maxwell V.A., Siracusa C., Were S.R. (2014). The Effect of Dog-Human Interaction on Cortisol and Behavior in Registered Animal-Assisted Activity Dogs. Appl. Anim. Behav. Sci..

